# Study on the Corrosion and Wear Mechanism of a Core Friction Pair in Methanol-Fueled Internal Combustion Engines

**DOI:** 10.3390/ma18091966

**Published:** 2025-04-25

**Authors:** Wenjuan Zhang, Hao Gao, Qianting Wang, Dong Liu, Enlai Zhang

**Affiliations:** 1 School of Mechanical and Electric Engineering, Sanming University, Sanming 365004, China; 20161134@fjsmu.edu.cn; 2 SINOMACH Intelligence Technology Co., Ltd., Guangzhou 510700, China; wqt@xmut.edu.cn; 3 ZYNP International Corporation, Industrial Cluster District, Mengzhou 454750, China; zynpliudong@163.com; 4 School of Mechanical and Automotive Engineering, Xiamen University of Technology, Xiamen 361024, China

**Keywords:** methanol-fueled engines, corrosion–wear coupling, high-chromium cast iron, corrosion-resistant gray cast iron, bench durability

## Abstract

With the global shift in energy structure and the advancement of the “double carbon” strategy, methanol has gained attention as a clean low-carbon fuel in the engine sector. However, the corrosion–wear coupling failure caused by acidic byproducts, such as methanoic acid and formaldehyde, generated during combustion severely limits the durability of methanol engines. In this study, we employed a systematic approach combining the construction of a corrosion liquid concentration gradient experiment with a full-load and full-speed bench test to elucidate the synergistic corrosion–wear mechanism of core friction pairs (cylinder liner, piston, and piston ring) in methanol-fueled engines. The experiment employed corrosion-resistant gray cast iron (CRGCI), high chromium cast iron (HCCI), and nodular cast iron (NCI) cylinder liners, along with F38MnVS steel and ZL109 aluminum alloy pistons. Piston rings with DLC, PVD, and CKS coatings were also tested. Corrosion kinetic analysis was conducted in a formaldehyde/methanoic acid gradient corrosion solution, with a concentration range of 0.5–2.5% for formaldehyde and 0.01–0.10% for methanoic acid, simulating the combustion products of methanol. The results showed that the corrosion depth of CRGCI was the lowest in low-concentration corrosion solutions, measuring 0.042 and 0.055 μm. The presence of microalloyed Cr/Sn/Cu within its pearlite matrix, along with the directional distribution of flake graphite, effectively inhibited the micro-cell effect. In high-concentration corrosion solutions (#3), HCCI reduced the corrosion depth by 60.7%, resulting in a measurement of 0.232 μm, attributed to the dynamic reconstruction of the Cr_2_O_3_-Fe_2_O_3_ composite passive film. Conversely, galvanic action between spherical graphite and the surrounding matrix caused significant corrosion in NCI, with a depth reaching 1.241 μm. The DLC piston coating obstructed the permeation pathway of formate ions due to its amorphous carbon structure. In corrosion solution #3, the recorded weight loss was 0.982 mg, which accounted for only 11.7% of the weight loss observed with the CKS piston coating. Following a 1500 h bench test, the combination of the HCCI cylinder liner and DLC-coated piston ring significantly reduced the wear depth. The average wear amounts at the top and bottom dead centers were 5.537 and 1.337 μm, respectively, representing a reduction of 67.7% compared with CRGCI, where the wear amounts were 17.152 and 4.244 μm. This research confirmed that the HCCI ferrite–Cr carbide matrix eliminated electrochemical heterogeneity, while the DLC piston coating inhibited abrasive wear. Together, these components reduced the wear amount at the top dead center on the push side by 80.1%. Furthermore, mismatches between the thermal expansion coefficients of the F38MnVS steel piston (12–14 × 10^−6^/°C) and gray cast iron (11 × 10^−6^/°C) resulted in a tolerance exceeding 0.105 mm in the cylinder fitting gap after 3500 h of testing. Notably, the combination of a HCCI matrix and DLC coating successfully maintained the gap within the required range of 50–95 μm.

## 1. Introduction

The global shift in energy infrastructure and the double carbon strategy has positioned methanol as a key alternative fuel for internal combustion engines due to its clean low-carbon properties [1,2,3]. As an alternative fuel with 50 wt% oxygen content, methanol has advantages such as a high octane number (RON 109) and high vaporization latent heat (1100 kJ/kg), reducing CO_2_ emissions from heavy-duty commercial vehicles by 10–15% over their service life. In marine power systems, methanol eliminates sulfur oxide emissions [4,5,6,7]. However, the strong polarity and hygroscopic nature of methanol fuel, along with the acidic byproducts generated during combustion such as formaldehyde (HCHO), methanoic acid (HCOOH), water, and hydrogen peroxide, can significantly degrade the physicochemical properties of lubricating oil. This deterioration poses serious challenges to the engine’s core components (cylinder liner, piston, and piston ring), which face the combined effects of corrosion and wear [6,8,9]. This coupled failure mechanism presents a major challenge to the durability of methanol engines.

Recent research has mainly focused on optimizing methanol fuel combustion [10,11,12] and reducing emissions [13,14,15]. These studies have suggested that by optimizing fuel formulation and innovating combustion modes, methanol fuel can significantly enhance engine performance and fuel economy and reduce emissions [16,17]. However, methanol fuel’s corrosive nature poses significant challenges to engine material durability. During combustion, methanol generates a formaldehyde intermediate product, which is converted into methanoic acid [9,18,19,20] through an oxidation reaction. This strong corrosive medium (pKa = 3.75) can induce the electrochemical corrosion of metal materials [21,22]. Cai et al. [23] employed simulated combustion solutions and corrosion fatigue tests. They found that cast iron cylinder liners exhibited higher corrosion rates in methanol environments compared with diesel or ammonia, characterized by loose α-Fe_2_O_3_ corrosion products and multi-source crack initiation. Kumar et al. [24] demonstrated through static immersion tests that pure methanol (M100) accelerated piston ring corrosion by a factor of 4–5 times compared with gasoline, exhibiting nonlinear kinetics due to self-inhibiting oxide layers. Furthermore, the diluting effect of methanol on lubricating oil altered the oil thickness and load-bearing capacity between the cylinder liner, piston ring, and piston friction pair, thereby impacting the tribological performance of the friction pair [25,26]. Xu et al. [27] identified methanol-induced lubricant dilution as a critical factor degrading oil film load capacity. However, laser texturing was found to reduce friction coefficients by 30–40%. Additionally, Su et al. [28] optimized MoDLC coatings through bias voltage modulation, achieving a wear rate of 5.2 × 10^−8^ mm^3^/(N·m) under methanol conditions. While these studies illuminated individual corrosion or wear mechanisms, systematic investigations into their synergistic effects on friction pairs have been insufficient. As a result, it is essential to explore the corrosion and wear of the core friction pair components of methanol engines in the context of combustion products derived from methanol fuel.

To address the aforementioned issue, this study investigated the corrosion–wear coupling mechanisms in methanol engines by integrating corrosion liquid concentration experiments with bench durability tests. The main study objective was to establish a material-coating synergy strategy that effectively mitigates electrochemical corrosion and mechanical wear in methanol combustion environments. We established three hypotheses. First, HCCI characterized by its Cr_2_O_3_-Fe_2_O_3_ dynamic passivation capability, can suppress acid-induced dissolution through the continuous reconstruction of the oxide film. Second, the oriented graphite architecture of microalloyed CRGCI, in conjunction with Sn/Cu addition, disrupts micro-galvanic corrosion pathways. Third, the amorphous carbon structure of DLC coatings provides dual protection by blocking formate ion penetration and reducing abrasive wear. Although previous studies have individually addressed methanol-induced corrosion [23,24] and lubricant-depleted wear [27,28], a critical gap remains in the current understanding of the synergistic degradation mechanisms under actual engine operating cycles. This study bridges that gap through the above innovative outlined approach.

This study focused on three types of cylinder liners (CRGCI, HCCI, NCI), two piston alloys (F38MnVS steel, ZL109 aluminum), and three piston ring coatings (DLC, PVD, CKS) under simulated methanol combustion conditions. Key innovations included clarifying the dynamic reconstruction of Cr_2_O_3_-Fe_2_O_3_ passive films in HCCI under acidic extremes, and the synergistic corrosion inhibition via Cr/Sn/Cu microalloying with oriented graphite in CRGCI. Through comprehensive long-term bench tests conducted at full speed and full load, the engineering applicability of the combination of HCCI (Cr > 25%) and DLC coating was verified, providing a theoretical basis for addressing the durability challenges faced by methanol engines.

## 2. Materials and Methods

### 2.1. Materials

**Figure 1:**
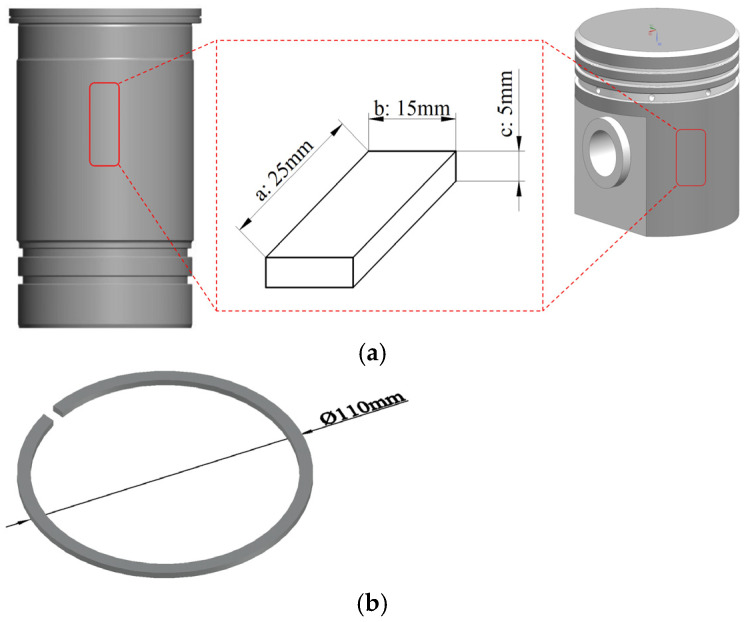
Cylinder liner, piston, and piston ring specimen geometries and specimen locations: (**a**) cylinder liner and piston (a represents the length, b represents the width, and c represents the thickness) and (**b**) piston ring.

The core friction pair consisted of a cylinder liner, piston, and piston ring. The materials used for the cylinder liner included corrosion-resistant gray cast iron (CRGCI), high-chromium cast iron (HCCI), and nodular cast iron (NCI), with the chemical compositions of these materials presented in Table 1. Table 2 details the chemical compositions of the two piston materials used in the test study, namely casting aluminum alloy (ZL109) and microalloyed medium carbon steel (F38MnVS). The selected piston ring surface coatings included a diamond-like carbon (DLC) coating, a composite coating of CrN and Cr_2_N prepared by physical vapor deposition (PVD), and a chromium-based Al_2_O_3_ ceramic composite plating known as a Chrom–Keramik–Schicht (CKS) coating. The geometric dimensions and sampling positions of the cylinder liner, piston, and piston ring test pieces are illustrated in Figure 1. Specifically, the geometric structures of the cylinder liner and piston were both cuboids measuring 25 × 15 × 5 mm (length × width × height). The piston ring was designed as a rectangular integral piston ring with a diameter of Φ110 mm.

### 2.2. Methods

The corrosion test followed the protocols outlined in the JB/T 7901 [29] and GB/T 21621 standards [30]. The corrosion conditions for each individual component, including the cylinder liner, piston, and piston ring, were established accordingly. A lubricating oil with a model designation of 10W30 was used, with W denoting winter, the number preceding W indicating the low-temperature fluidity of the engine oil, and the number following W representing the kinematic viscosity of the oil at 100 °C. Table 3 presents the three different proportions of corrosion solutions employed in the test, which consisted of formaldehyde, methanoic acid, and lubricating oil. The concentration ranges of formaldehyde (0.5–2.5 wt%) and methanoic acid (0.01–0.10 wt%) in corrosion solutions were determined based on analyses of methanol combustion products and engine exhaust measurements [1,7]. Methanol combustion under full-load conditions generates formaldehyde concentrations of 1.2–2.8% in blow-by gases [4]. In contrast methanoic acid accumulation in lubricants reaches 0.05–0.12% after 500 h of operation [23]. The selected gradients (#1 to #3) were used to simulate scenarios ranging from mild cold-start transients (#1: 0.5% HCHO + 0.01% HCOOH) to severe high-load deterioration (#3: 2.5% HCHO + 0.10% HCOOH). The effects of lubricant dilution were considered by maintaining a base oil content greater than 97%, which reflects typical oil film contamination rates (3–5%) in methanol engines [26]. This methodology ensures that the corrosion solutions effectively replicate both transient and steady-state exposure conditions encountered by friction pairs. Each test piece of the cylinder liner, piston, and piston ring was soaked and cleaned in diluted hydrochloric acid for 3 min. After removal, the surface of each test piece was rinsed with absolute ethanol to eliminate any residual dilute hydrochloric acid. The pieces were then dried using hot air for 30 min. Immediately afterward, the specimens were weighed and the weight was recorded as M_1_ (weight of the test piece before corrosion) using an electronic balance. Then, a round-bottom flask was filled with 250 mL of corrosive liquid and placed in a constant temperature water bath, which was heated to 45 °C. Once the temperature was reached, the test piece was carefully slid along the inner wall into the flask and soaked for 180 min before being removed. The surface of the test piece was immediately rinsed with absolute ethanol to remove any residual corrosive liquid. After drying with hot air for 30 min, the weight was recorded on an electronic balance as M_2_ (weight of the test piece after corrosion). Next, one side of the 25 × 5 mm surface of the test piece was ground and polished. The corrosion depth (H) values of the cylinder liner test pieces, consisting of CRGCI, HCCI, and NCI, were calculated using Equation (1). In Equation (1), M_1_ and M_2_ denote the weights before and after corrosion (mg), respectively, and ρ denotes the material density (mg/mm^3^), with values of CRGCI at 7.25, NCI at 7.10, and HCCI at 7.60. The term (ab + ac + bc) × 2 represents the surface area (mm^2^), as illustrated in Figure 1a for lengths a, b, and c. Each material type underwent three parallel tests to ensure repeatability. The corrosion depth values exhibited a standard deviation of ≤5% across replicates, indicating minimal variability. Data were expressed as average ± standard deviation. The average corrosion depth was recorded as the final test result. Potential uncertainties, including minor temperature fluctuations (±1 °C) in the water bath and variations in specimen surface roughness (Ra ≤ 0.8 μm), were controlled within acceptable limits to minimize their impact on corrosion kinetics.

(1) $$H=\frac{M_{1}-M_{2}}{\rho \times \left[\left(ab+ac+bc\right)\times 2\right]}\times 10^{6}$$

The bench durability test utilized an M100 methanol spark-ignition in-line 6-cylinder engine, which was run-in prior to testing, with the basic test parameters of the engine presented in Table 4. Full-speed, full-load, long-term bench tests were conducted in accordance with the GB/T19055-2024 standard [31]. To ensure repeatability, we performed three independent tests for each engine configuration (11 and 13 L), with the coolant temperature stabilized at 85 ± 2 °C and the lubricant pressure maintained at 4.0 ± 0.1 bar. Measurement uncertainties, including ±2.0 μm for wear depth (measured using a Hommel T8000 profiler, Villingen-Schwenningen, Germany) and ±0.002 mm for dimensional parameters (using an RA300 form line instrument), were quantified through calibration against certified reference standards. Tests were performed using 11 and 13 L engines to assess the wear and fit of the cylinder liner, piston, and piston ring within the power assembly. Moreover, minor variations in the engine load (±1.5%) and ambient humidity (45–55%) were recorded but were deemed negligible due to their limited effect on wear trends. The lubricating oil used for the tests was 10W30. Following the tests, the power assembly of the engine was disassembled to analyze and evaluate the wear of the cylinder liner, the fit clearance between the piston and the cylinder liner, and the dimensional parameters of the piston ring.

### 2.3. Characterization Equipment

The corrosion immersion test was conducted on a test piece using a constant temperature water bath (HH-4, LICHEN, Shanghai, China). An electronic balance (MS105DU, Mettler Toledo MTCN Limited Company, Hong Kong, China) was used to weigh the test piece before and after the corrosion test. The metallographic microstructure of the material was observed using a Leica metallographic microscope (DM2000, Wetzlar, Germany), and a roughness profiler (T8000, Hommel, Germany) was employed to measure the corrosion and wear depth of the test piece. The diameter of the piston group was measured using a form line measuring instrument (RA300, Shanxi Wale Mechanical and Electrical Technology Co., Ltd., Xi’an, China), in conjunction with an outside micrometer (293, Best Instrument Co., Ltd., Suzhou, China). Measurements were taken three times at the designated position, and the average value was recorded. In addition, the inside diameter of the cylinder liner was measured using an inside diameter micrometer (468, Hangzhou Round Testing Instrument Co., Hangzhou, China), and three measurements were taken at the corresponding position of the piston to calculate the clearance fit value between the cylinder liner and the piston.

## 3. Results and Analyses

### 3.1. Corrosion Test

#### 3.1.1. Cylinder Liner Corrosion Analysis

**Figure 2:**
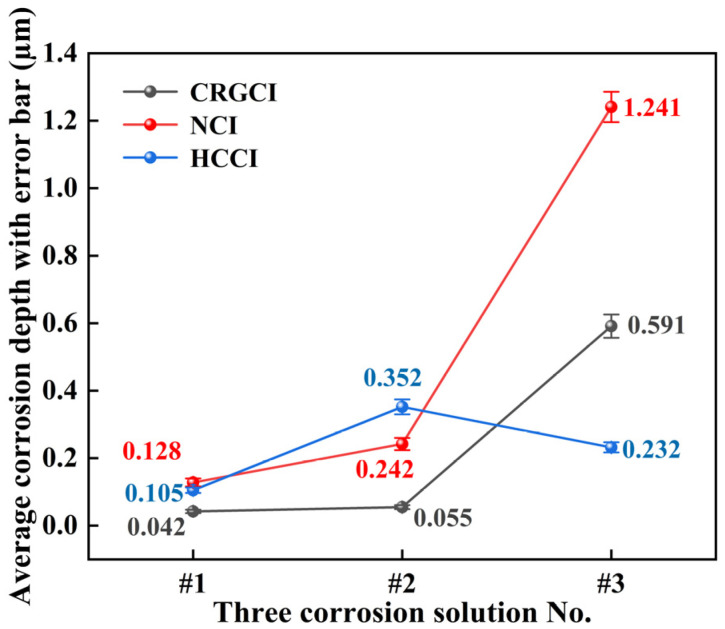
Average corrosion depths with error bar for CRGCI, NCI, and HCCI specimens in the three corrosive solutions.

Table 5 and Figure 2 present the average corrosion depths with the standard deviation and error bar of the CRGCI, HCCI, and NCI cylinder liner specimens exposed to the three types of corrosive solutions. As the concentrations of formaldehyde and methanoic acid increased, the corrosiveness of these liquids toward the specimens gradually intensified. The average corrosion depths of CRGCI in the three corrosive solutions were measured as 0.042, 0.055, and 0.591 µm. Notably, the corrosion depth was lowest in corrosive solutions #1 and #2. With increasing concentrations of formaldehyde and methanoic acid in the corrosive liquid, the corrosion depth of NCI increased from 0.128 to 1.241 µm. In corrosive solution #3, the corrosion depth sharply increased, reaching 2.1 times that of CRGCI. The average corrosion depths of HCCI in the three corrosive solutions were 0.105, 0.352, and 0.232 µm, and in corrosive solution #3, which had high formaldehyde and methanoic acid content, the corrosion depth was the lowest compared with the other two materials.

**Figure 3:**
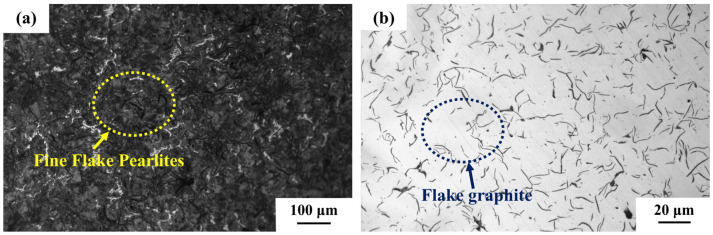
Metallographic organization of CRGCI: (**a**) matrix and (**b**) flake graphite.

As shown in Figure 2, CRGCI exhibited optimal corrosion resistance in low-concentration corrosion solutions #1 and #2, with corrosion depths measuring 0.042 ± 0.005 and 0.055 ± 0.006 µm, respectively. The low standard deviations observed for CRGCI (e.g., ±0.005 μm in solution #1) confirmed the reproducibility of its superior corrosion resistance under low-concentration conditions. This was primarily attributed to the microalloying effect of 0.2–0.8% chromium elements within its pearlite matrix, as shown in Figure 3a. In the corrosion environment of low-concentration methanoic acid (0.01–0.02%) and formaldehyde (0.5–1.5%), an oxide film predominantly composed of Fe_3_O_4_ preferentially formed on the surface of the Fe–Cr solid solution. Although its homogeneous density was lower than that of Cr_2_O_3_, the synergistic effect of Sn (0.05–0.2%) and Cu (0.1–0.5%) in the composition effectively inhibited the anodic dissolution reaction Fe→Fe^2+^ + 2e^−^. Furthermore, the directional distribution of flake graphite (Figure 3b) mitigated the degree of continuous matrix damage and reduced the formation of local corrosion microcells. However, as the concentration of the corrosion solution increased to #3 (0.1% methanoic acid + 2.5% formaldehyde), formaldehyde and methanoic acid created an acidic environment in the solution. Methanoic acid (HCOOH), as a weak acid, could partially ionize to generate H^+^ and formate ion (HCOO^−^), according to Formula (2):

HCOOH⇌H^+^ + HCOO^−^. (2)

The increase in H^+^ concentration led to the acidolysis reaction of the Fe_3_O_4_ film, as represented by Formula (3), which resulted in a sudden increase in corrosion depth to 0.591 μm:

Fe_3_O_4_ + 8H^+^→Fe^2+^ + 2Fe^3+^ + 4H_2_O. (3)

**Figure 4:**
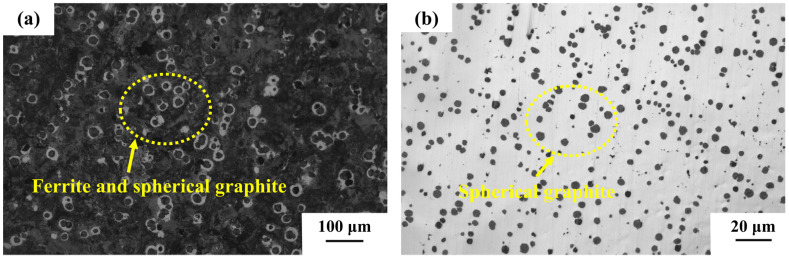
Metallographic organization of NCI: (**a**) matrix and (**b**) spheroidal graphite.

The primary components of NCI were Fe, C, Si, and Mg. The graphite present existed in spherical form, as illustrated in Figure 4, and it also contained a small amount of Cr and other alloying elements. Due to the low chromium content, ductile iron could not form a stable Cr_2_O_3_ film, unlike HCCI. Instead, it primarily generated Fe_2_O_3_ or Fe_3_O_4_ [23]. These oxide films provided inadequate protection, leading to a significant increase in corrosion depth as the concentration of the corrosive liquid increased. Furthermore, spherical graphite in ductile iron could function as a cathodic phase, exacerbating local galvanic corrosion. The overall corrosion mechanism of ductile iron in the presence of formaldehyde and methanoic acid is shown in Formulas (4) and (5):

Fe+2HCOOH→Fe^2+^ + 2HCOO^−^ + H_2_, (4)

4Fe + 3O_2_ + 6HCHO→2Fe_2_O_3_ + 6HCOOH. (5)

The corrosion kinetics of HCCI showed nonlinear characteristics. The average corrosion depths in the #1 and #2 corrosion solutions were 0.105 and 0.352 μm, respectively, which fell to 0.232 μm in corrosion solution #3. As previously mentioned, CRGCI and NCI exhibited increased corrosion depth in the #3 corrosive solution due to the acid concentration rise. This increase triggered the acidolysis reaction of the Fe_3_O_4_ oxide film on the specimen surface (see Equation (3)), thus diminishing its protective effect. However, HCCI demonstrated reduced corrosion depth under high-concentration #3 conditions, owing to the synergistic effects of dynamic passivation, selective precipitation, and microstructural stability. The corrosion mechanism was attributed to the 25–30% high chromium content, which promoted the formation of a continuous Cr_2_O_3_ passive film on the test piece surface. This formation followed the reaction 2Cr + 3H_2_O→Cr_2_O_3_ + 6H^+^ + 6e^−^ [32]. Additionally, formaldehyde (HCHO) was oxidized to form methanoic acid or other intermediate products in an acidic environment, which further increased the solution’s acidity. Chromium (Cr) was oxidized in an acidic environment to generate chromium ions (Cr^3+^), as shown in the reaction in Equation (6):

Cr→Cr^3+^ + 3e^−^. (6)

**Figure 5:**
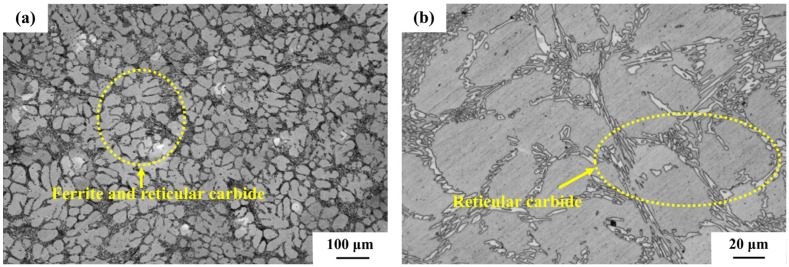
Metallographic organization of HCCI: (**a**) matrix and (**b**) reticular carbide.

In low-concentration corrosion solution #1, the Cr_2_O_3_ passivation film remained stable through dynamic equilibrium. In medium-concentration solution #2, the HCOO^−^ generated by the ionization of methanoic acid formed a complex with Cr^3+^, which triggered local pitting and led to an increase in corrosion. In solution #3, the increased H^+^ concentration sped up the dissolution of metastable Fe-rich oxides, including Fe_3_O_4_, facilitating the dynamic reconstruction of a denser Cr_2_O_3_-Fe_2_O_3_ composite passive film. This film exhibited a reduced defect density and improved chemical stability owing to covalent Cr^3+^-O bonding, which effectively obstructed the permeation of corrosive ions [33]. Simultaneously, the increase in methanoic acid concentration aided in the formation of Fe(HCOO)_2_ precipitate from Fe^2+^ and HCOO^−^. This reaction, in turn, inhibited the anodic reaction of corrosion and reduced the corrosion depth. In addition, the electrochemical potential difference between the chromium-alloyed ferrite matrix and (Fe, Cr) carbides in HCCI was minimized, thereby mitigating micro-galvanic corrosion [34]. Contrastingly, the spherical graphite in NCI acted as a cathodic phase, which intensified localized galvanic corrosion at the graphite–matrix interface. The interconnected (Fe-Cr) carbide network in HCCI (Figure 5) further enhanced corrosion-wear resistance, thus preserving the integrity of the passive film under corrosion–wear coupling conditions. These mechanisms collectively accounted for the reduced HCCI corrosion depth in high-concentration solution #3 compared with that of CRGCI and NCI.

CRGCI exhibited a slow corrosion rate and minimal corrosion depth in environments with low concentrations of formaldehyde and methanoic acid (#1 and #2). Under actual working conditions, when the methanol engine was operational, the surface of the cylinder liner was coated with a lubricating oil film, which served to isolate the corrosive medium produced by the acidic byproducts of methanol combustion. In addition, the high-temperature environment (>100 °C) accelerated the formation of the lubricating oil film and promoted the partial densification of the iron oxide layer, indirectly enhancing short-term corrosion resistance. Due to its balanced performance and cost advantages, CRGCI could serve as an ideal material for cylinder liners.

#### 3.1.2. Piston Corrosion Analysis

**Figure 6:**
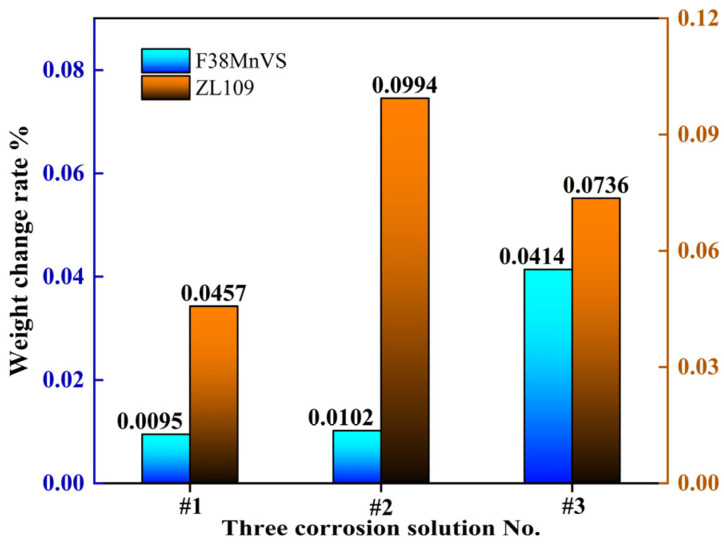
Weight change rates of F38MnVS and ZL109 piston in corrosive solution.

Table 6 presents the average corrosion depths observed during the corrosion tests of the F38MnVS steel and cast aluminum alloy ZL109 pistons. The F38MnVS steel primarily experienced reduction in depths in the three types of corrosion solutions, recorded at 0.155 ± 0.02, 0.166 ± 0.03, and 0.676 ± 0.04 µm. Notably, the reduction in depths due to corrosion significantly increased with a higher concentration of formaldehyde and methanoic acid in the corrosion solution. The corrosiveness of corrosion solution #3 toward the steel was the highest, indicating that the dissolution effect of the high-concentration methanoic acid formaldehyde corrosion medium on the steel matrix was more pronounced. The cast aluminum alloy ZL109 exhibited increases in depths after the corrosion test, with gains of 0.746 ± 0.05, 1.622 ± 0.05, and 1.201 ± 0.04 µm. The F38MnVS steel primarily exhibited weight loss in three types of corrosion solutions, with losses recorded at 1.4, 1.5, and 6.1 mg, respectively. In contrast, the ZL109 demonstrated weight gains of 2.3, 5.0, and 3.7 mg following the corrosion tests. As illustrated in Figure 6, the weight loss change rates of the steel piston specimens were 0.0095%, 0.0102%, and 0.0414%, while the weight gain change rates of the aluminum piston specimens were 0.0457%, 0.0994%, and 0.0736%. In general, the weight change rate of the aluminum piston specimens was greater than that of the steel pistons. The corrosion data are shown in Table 6 and Figure 6, indicating that the corrosion resistance of the aluminum piston in the methanol engine was inferior to that of the steel piston.

The corrosion behavior of the F38MnVS steel piston in formaldehyde/methanoic acid solutions was characterized by a localized electrochemical dissolution mechanism [21,22]. In the presence of formaldehyde/methanoic acid, the iron matrix functioned as the anode, undergoing a dissolution reaction represented by Fe→Fe^2+^ + 2e^−^. Concurrently, the H^+^ ions in the corrosion solution participated in a hydrogen evolution reaction at the cathode, described by 2H^+^ + 2e^−^→H_2_↑. As the concentration of the corrosion solution increased from #1 to #3, the acidity rose, which accelerated the oxidation of Fe^2+^. This reaction could be expressed as 4Fe^2+^ + O_2_ + 6H_2_O→4FeOOH + 8H^+^. The resulting FeOOH was subsequently transformed into soluble Fe^3+^ in the more acidic environment, leading to the formation of a porous non-protective oxide film. The sulfur content (0.035–0.075 wt%) in the steel led to the formation of MnS inclusions, which functioned as micro-cathodes, thus accelerating pitting corrosion at the interfaces between the inclusions and the matrix [35]. As a result, the weight loss of the steel specimens in the #3 corrosion solution reached 6.1 mg, representing a 336% increase compared with that in corrosion solution #1.

Conversely, the ZL109 cast aluminum alloy exhibited dynamic passivation–dissolution corrosion [36]. Initially, the aluminum surface formed a protective Al_2_O_3_ layer through the reaction 4Al + 3O_2_→2Al_2_O_3_. This precipitated the Al_2_O_3_ passivation film adhering to the surface of the test piece, resulting in an increase in mass. In the experiment, the weight gains observed for samples #1 and #2 were 2.3 and 5.0 mg, respectively. However, the acidic medium (H^+^) continuously dissolved this oxide film through the reaction Al_2_O_3_ + 6H^+^→2Al^3+^ + 3H_2_O, which exposed the underlying matrix to further corrosion. The regenerated Al^3+^ ions hydrolyzed to form Al(OH)_3_ precipitates through the reaction Al^3+^ + 3H_2_O→Al(OH)_3_↓ + 3H^+^, and subsequently reacted with formate ions (HCOO^−^) to produce insoluble aluminum formate complexes (Al(HCOO)_3_↓). Although the Al_2_O_3_ film was continuously regenerated, high H^+^ concentrations in solution #3 sped up Al^3+^ dissolution (Al→Al^3+^ + 3e^-^), reducing solid deposition and explaining the lower weight gain (3.7 mg in solution #3 versus 5.0 mg in solution #2).

#### 3.1.3. Piston Ring Corrosion Analysis

**Figure 7:**
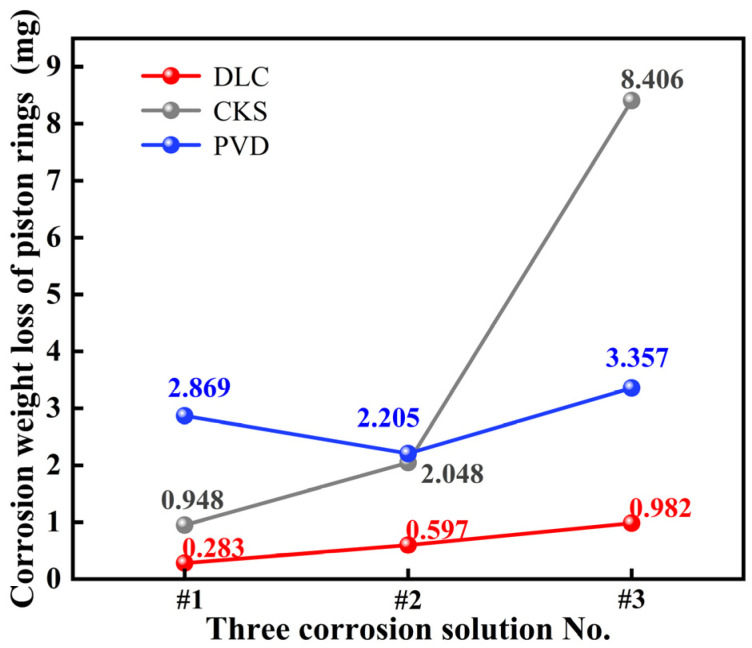
Corrosion weight loss of DLC-, CKS-, and PVD-coated piston rings in three corrosive solutions.

Figure 7 illustrates the corrosion weight loss of coated rings (DLC-, CKS-, and PVD-coated) with identical geometric specifications but different surface coatings when immersed in three distinct corrosive fluids. The test data indicated that the DLC coating exhibited the lowest weight loss in corrosion solutions #1, #2, and #3, measuring 0.283, 0.597, and 0.982 mg, respectively. By contrast, the CKS coating exhibited slightly higher weight loss than the DLC coating in low-concentration corrosion solutions #1 and #2, with measurements of 0.948 and 2.048 mg, respectively. However, the weight loss of the CKS coating increased sharply to 8.406 mg in high-concentration corrosion solution #3. The PVD coating demonstrated the highest weight loss in corrosion solutions #1 and #2, with values of 2.869 and 2.205 mg, respectively. However, in the high-concentration #3 corrosion solution, the weight loss fell between that of the DLC and CKS coatings, measuring 3.357 mg.

The weight loss of the DLC coating in the three corrosion solutions was significantly lower than that of the PVD and CKS coatings, and its excellent corrosion resistance was attributed to the physical shielding effect of the amorphous carbon structure. The dense carbon layer inhibited contact between the corrosive medium, H^+^ and formate ion (HCOO^−^), and the substrate metal, effectively hindering the penetration of H^+^ generated by methanoic acid ionization. However, in the high-concentration #3 corrosion solution, the weight loss of the DLC coating increased by 64.5% compared with that in the #2 corrosion solution. This increase was due to the increasing concentration of methanoic acid and formaldehyde, which triggered a micro-region dissolution reaction (Fe + 2H^+^→Fe^2+^ + H_2_↑) in the iron substrate of the test piece, leading to a gradual increase in the corrosion weight loss of the DLC coating. The weight loss of the PVD coating in low-concentration corrosion solutions #1 and #2 was greater than that of the DLC coating. This phenomenon could be attributed to the formation of micron-sized channels at the grain boundaries in the CrN phase, which accelerated the penetration of the corrosion solution. In addition, the micro-galvanic effect generated by the CrN phase (potential of +0.74 V) and the Cr_2_N phase (potential of −0.42 V) enhanced the anodic dissolution of the Cr_2_N phase at the grain boundaries, represented by the reaction Cr→Cr^3+^ + 3e^−^ [37]. Conversely, in the #3 high-concentration corrosion solution, the weight loss was lower than that of the CKS coating. This could be explained by the fact that H^+^ ions promoted the dynamic reconstruction of the Cr_2_O_3_ passivation film, as indicated by the reaction 4CrN + 3O_2_ + 6H_2_O→2Cr_2_O_3_ + 4NH_3_↑ [32]. The corrosion behavior of the CKS coating exhibited a significant dependence on concentration. The weight loss observed in the #3 corrosion solution was 8.406 mg, representing a 310% increase compared with the weight loss in corrosion solution #2. This phenomenon could be attributed to the hydrolysis reaction of the Al_2_O_3_ ceramic phase under acidic conditions, which produced a loose coating structure. Once the Cr matrix was exposed, a localized corrosion micro-battery formed. The potential difference between the Cr-based anode and the residual Al_2_O_3_ cathode further accelerated the dissolution of the anode.

The superior corrosion resistance of DLC coatings indicates their potential applicability to other engine components exposed to methanol-derived corrosive environments, including valve stems, fuel injectors, and camshaft bearings [38]. However, extending DLC coatings to cover the entire piston surface poses several challenges. First, while the amorphous carbon structure effectively blocks corrosive ions, large-area deposition on complex geometries (e.g., piston skirts or pin bosses) could result in localized coating delamination due to the thermal expansion mismatch between the DLC layer (coefficient of thermal expansion (CTE ≈ 4–6 × 10^−6^/°C)) and the steel/aluminum substrate (CTE: 12–24 × 10^−6^/°C) [39]. Additionally, the high hardness (15–40 GPa) and low ductility of DLC coatings could worsen abrasive wear on softer mating surfaces, including aluminum cylinder liners. For full-piston applications, hybrid strategies include combining DLC-coated rings with selectively hardened piston skirt regions or optimizing interlayer adhesion through plasma-enhanced chemical vapor deposition (PECVD). Such strategies could mitigate these drawbacks while maintaining corrosion resistance [40].

### 3.2. Bench Durability Test

#### 3.2.1. 11 L Engine Bench Test

**Figure 8:**
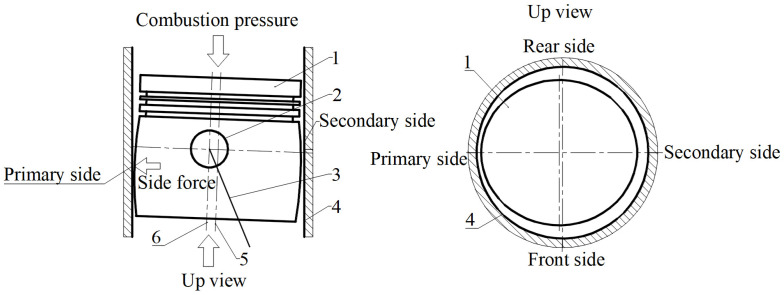
Schematic diagram of the cylinder liner affected by piston movement on the front, rear, primary, and secondary thrust sides. 1—piston, 2—piston pin, 3—piston connecting rod, 4—cylinder liner, 5—piston center, 6—piston pin center.

A 10W30 special lubricating oil for methanol engines was used to conduct a 1000 h durability test on an 11 L methanol engine. The selected friction pair materials included CRGCI for the cylinder liner, and F38MnVS steel for the piston and piston rings, where one ring had a DLC coating, two rings had a PVD coating, and the oil ring had a DLC coating. The wear data obtained from the cylinder liner durability test are presented in Supplementary Table S1. Figure 8 displays schematic diagrams of the front side, rear side, primary push side, and secondary push side positions influenced by piston movement in the cylinder liner.

**Figure 9:**
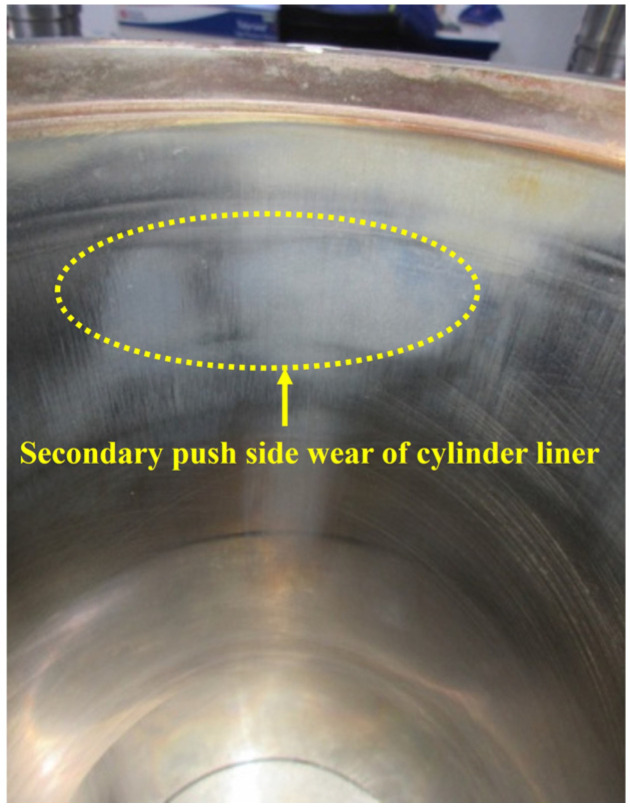
Wear surface of the liner bore.

The data in Table S1 indicate that the wear depth of the cylinder liner pair in the secondary push side direction ranged from 17.403 to 32.684 μm. The wear depth exceeded 17 μm, highlighting the characteristics of a typical high wear area. Notably, the wear depth of the #1 cylinder liner pair on the secondary push side reached 32.684 μm, which was 3–13 times greater than that of its primary push, rear, and front sides. The wear depth of the primary push side of the six cylinder liners exhibited significant fluctuations, ranging from 7.1 to 23.182 μm, with the maximum value recorded for the #3 cylinder liner at 23.182 μm. By contrast, the wear depth of the front and rear sides of the six cylinder liners was generally below 10 μm, with only the rear side of the #6 cylinder liner sample reaching 9.61 μm. Consequently, the front and rear sides were categorized as low wear areas of the cylinder liner specimens. As illustrated in Figure 9, the wear amount of the cylinder liner specimen in the top dead center (TDC) area was relatively substantial following the durability test.

During durability tests, the surface wear of the inner hole of the cylinder liner was influenced by the synergistic effects of mechanical and corrosive wear, where the pressure on the combustion side was concentrated on the secondary push side. The strength of the lubricating oil film was diminished due to the hygroscopic nature of methanol [41], which resulted in the failure of boundary lubrication. The flake graphite that detached from the cylinder liner matrix, along with debris generated by the DLC coating of the piston ring, contributed to abrasive wear on the inner wall of the cylinder liner. Concurrently, acidic substances such as methanoic acid produced by methanol combustion induced micro-cell corrosion of the pearlite matrix in the gray cast iron cylinder liner [42]. The corrosion products were mechanically removed by the reciprocating piston ring, creating a cycle of corrosion and material loss. In addition, the stress concentration of flake graphite further accelerated material loss on the secondary push side.

#### 3.2.2. 13 L Engine Bench Test

The durability test was conducted using a 13 L methanol engine, with 10W30 lubricating oil. For wear comparison, two types of friction pair materials were selected, specifically, CRGCI and HCCI for the cylinder liner. The piston was constructed from F38MnVS steel, while the piston ring assembly included one DLC ring, two PVD chromium-plated rings, and an oil ring with a DLC coating. The durability test was performed over a duration of 1500 h, with the wear amounts for the CRGCI and HCCI cylinder liners presented in Tables S2 and S3, respectively. Table S2 details the wear amounts of the CRGCI cylinder liners. Notable wear depths were observed at the TDC of the front side, secondary push side, rear side, and primary push side of the test cylinder liners’ inner surfaces. Specifically, the wear depths were recorded as follows: 43.377 µm on the front side of #5, 47.619 µm on the secondary push side of #3, 44.935 µm on the rear side of #4, 40.26 µm on the primary push side of #3, and 60 µm on the primary push side of #6. The average wear amount at the TDC of the six CRGCI cylinder liners was 17.152 µm, while the average wear amount at the bottom dead center (BDC) was 4.244 µm, as shown in Table 7.

**Figure 10:**
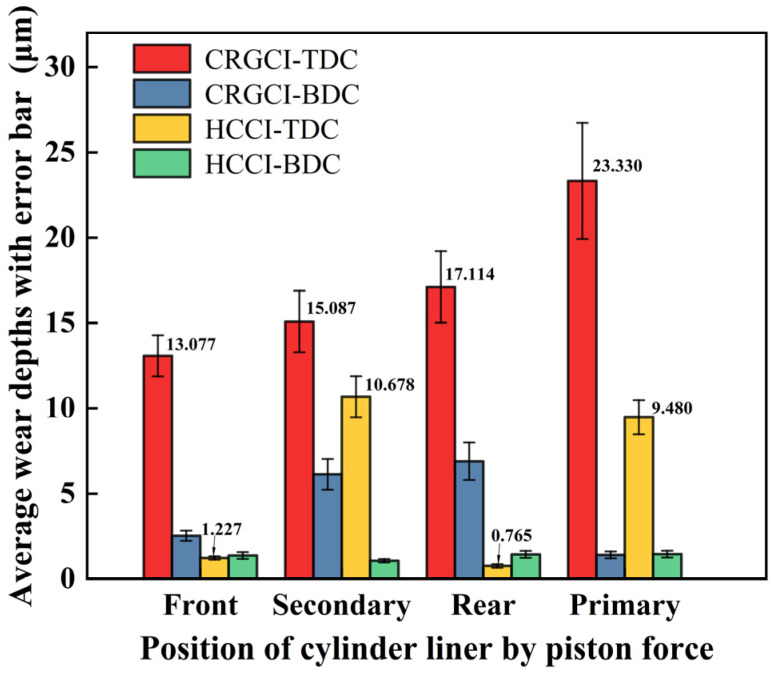
Comparison of CRGCI and HCCI average wear depths with error bar at TDC and BDC.

**Figure 11:**
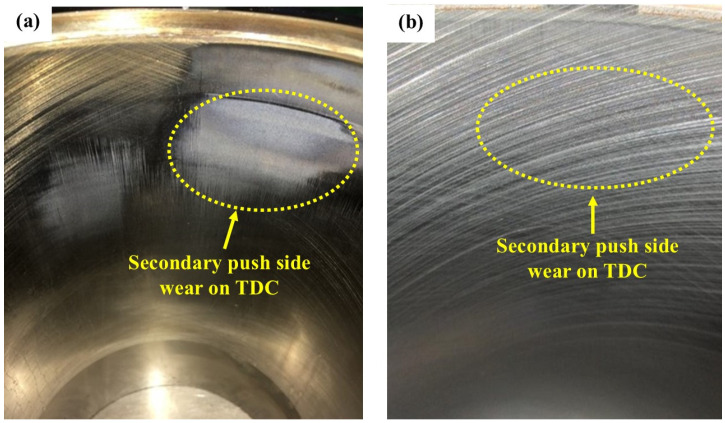
CRGCI and HCCI liners after 1500 h of bore wear: (**a**) CRGCI and (**b**) HCCI.

Table S3 presents the wear amounts of HCCI cylinder liners, indicating that the primary wear of the cylinder liner occurred in the TDC area on both the secondary push and primary push sides. The wear at the TDC of the secondary push side ranged from 8.225 to 12.727 µm, while the wear at the TDC of the primary push side ranged from 5.541 to 11.082 µm. Table 7 presents the average wear amount at the TDC across the six HCCI cylinder liners, which was 5.537 µm. Contrastingly, the average wear amount at the BDC was 1.337 µm, which is less than 1.6 µm. Figure 10 presents a comparison of the average wear amounts at the TDC and BDC of the CRGCI and HCCI cylinder liners. The average wear depths of the CRGCI cylinder liner at the front side, secondary push side, rear side, and primary push side of the TDC area were 13.077, 15.087, 17.114, and 23.330 µm, respectively. By contrast, the average wear depths of the HCCI at the same positions in the TDC area were 1.227, 10.678, 0.765, and 9.480 µm. Similarly, the average wear depth at the BDC of the CRGCI specimen was greater than that of the HCCI specimen. Overall, the average wear depth at both the TDC and BDC of the CRGCI was three times that of the HCCI. Figure 11 illustrates the TDC area of the #3 cylinder liner pair on the secondary push side, composed of CRGCI and HCCI. The wear on the inner wall of the gray cast iron cylinder liner was notably severe, with certain areas exhibiting a polished texture that appeared bright. By contrast, the texture on the inner wall of the HCCI test piece in the same region remained relatively well defined. This observation indicated that HCCI demonstrated superior wear resistance at both the TDC and BDC, and at various other positions. This advantage was particularly pronounced in high wear areas, such as the primary push side and secondary push side. Furthermore, the combination of a DLC-coated piston ring and HCCI significantly reduced wear depth. The average wear amount at the TDC was 5.537 ± 0.6 μm, representing a reduction of 67.7% compared with CRGCI (17.152 ± 2.1 μm). Similarly, at the BDC, HCCI exhibited 1.337 µm wear, 68.5% lower than CRGCI (4.244 µm). However, the excellent performance of HCCI + DLC was accompanied by trade-offs. Namely, the high chromium content (25–30%) in HCCI increased raw material costs by approximately 40% compared with traditional gray cast iron [43]. In contrast the DLC coating, applied through PVD technology, raised manufacturing costs by about 15% [44]. Due to the hardness of HCCI (450–600 HV), specialized tools are required for machining, resulting in a 20–30% increase in machining time [43]. The thermal expansion mismatch between HCCI (CTE: 10.5 × 10^−6^/°C) and steel pistons (12–14 × 10^−6^/°C) also necessitates a precise clearance design to avoid thermal stress under cyclic loading. Despite these challenges, HCCI cylinder liners demonstrated superior wear resistance. The liners considerably reduced wear during contact with DLC piston rings, thus extending the service life of engine components [45,46,47]. To adapt this configuration for smaller scale engines (e.g., automotive or auxiliary units), designers should evaluate trade-offs between HCCI’s machining costs and durability gains. For instance, the selective application of HCCI to high-stress regions (e.g., TDC zones) paired with standard materials elsewhere could optimize cost-effectiveness without compromising reliability. This enhancement is critically important for methanol engines.

In CRGCI, flake graphite (lower potential) and the pearlite matrix (higher potential) formed local microcells, which accelerated the matrix’s anodic dissolution. In the acidic corrosion environment of methanol combustion products, the matrix surrounding the graphite corroded first, leading to a rough surface on the material. For example, the front side of #5 measured 43.377 μm. After the flake graphite peeled off, free abrasive particles formed. Meanwhile, on the primary push side of 6#, the depth was 60 μm, which exacerbated the wear of the inner surface of the cylinder liner. During the testing process, the iron-based oxide film on the surface of the CRGCI repeatedly broke, allowing the acidic corrosion medium to invade the matrix and accelerate material loss. Consequently, the average wear depths at the TDC and BDC points were 17.152 and 4.244 μm, respectively. These values were significantly deeper than the average wear depths at the TDC and BDC points of HCCI, which measured 5.537 and 1.337 μm, respectively.

The metallographic organization of HCCI is illustrated in Figure 5 of Section 3.1.1. The matrix consisted of ferrite and reticular carbide, which effectively mitigated the abrasive wear issue caused by graphite exfoliation. The reticular carbide ((Fe, Cr)_4_C and (Fe, Cr)_23_C_6_) in HCCI exhibited high hardness and formed a rigid skeletal structure, thereby effectively resisting the indentation and cutting of abrasive grains resulting from wear. High chromium ferrite (Cr > 12.5%) generated a dense passive film (Cr_2_O_3_) through the solid solution of chromium, significantly enhancing the corrosion resistance of the matrix. Concurrently, the negative potential of ferrite transformed into a positive potential, with the potentials of the carbide phases ((Fe, Cr)_4_C and (Fe, Cr)_23_C_6_) approaching those of the ferrite matrix. This transformation eliminated the micro-battery effect and inhibited electrochemical corrosion. The wear observed at the TDC and BDC points during testing was relatively low, indicating that, in low-stress areas, the stability of the passive film played a crucial role in reducing corrosion wear on the inner surface of HCCI.

### 3.3. Piston and Piston Ring Inspection and Analysis

A 3500 h durability test was conducted using an 11 L methanol engine. The selected components included 10W30 lubricating oil, a CRGCI cylinder liner, an F38MnVS steel piston, and a piston ring configuration consisting of one DLC-coated ring, two PVD chrome-plated rings, and one DLC oil ring. After the durability test, the fit clearance between the steel piston and the gray cast iron cylinder liner was measured, along with the dimensions of the piston rings.

#### 3.3.1. Piston and Cylinder Liner Clearance

**Figure 12:**
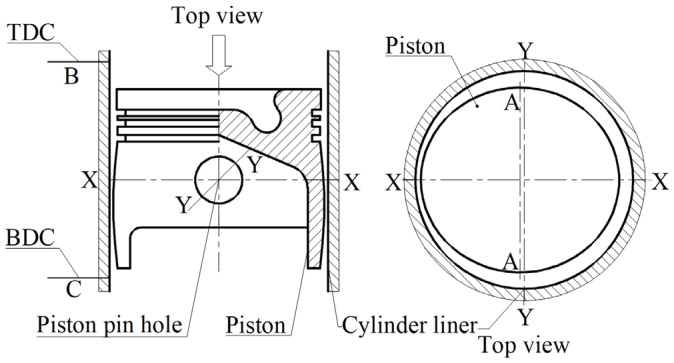
Piston and cylinder liner clearance detection position.

Figure 12 presents the detection positions of the fit clearance between the piston and the cylinder liner, with position A marking the location where the diameter variation of the piston group was the most significant, and positions B and C representing the detection points when the piston reached the TDC and BDC during its movement within the cylinder liner. Due to the uneven thickness of the piston skirt, the metal in the piston pin boss hole region was thicker, resulting in greater thermal expansion. Consequently, the deformation along the axis of the piston pin hole (Y-Y) was more pronounced than in the direction perpendicular to the piston pin hole (X-X). Therefore, the cylinder clearance was primarily assessed by the change in diameter in the Y-Y direction. At points B and C, corresponding to the TDC and BDC of the piston movement, respectively, the outer diameter of the piston at point A was measured, along with the inner diameter of the cylinder liner. This enabled the determination of the dimensional changes in the piston and cylinder liner diameters. Subsequently, the cylinder fitting clearance was calculated using Formula (7):

Clearance = Δ cylinder dimension − Δ piston skirt dimension.(7)

Table S4 details the diameter size changes of cylinder liners #1–6 in the Y-Y direction, ranging from 9 to 26 μm, with a positive value indicating an increase in diameter size change. The technical requirement specified a range of 0–25 μm. Notably, the diameter size change in section A of cylinder liner #1 was 26 μm, which exceeded the technical requirement by 1 μm. Table 8 shows the range of diameter change in the Y-Y direction for piston group parts #1 to #6 was between −80 and −75 μm, with the negative sign indicating a decrease in diameter. The technical requirement for diameter change was between −70 and −50 μm. The observed decrease in the diameter change in the piston skirt exceeded the specified technical requirements. After 3500 h of endurance testing, the matching clearance between the cylinder liner and piston group parts #1–6 ranged from 84 to 105 μm. Notably, the matching clearance between the cylinder liners of parts #1 and #5 and their respective pistons exceeded the maximum allowable value of 95 μm, as outlined in the technical requirements. In practical terms, an excessive clearance (e.g., 105 μm) disrupts the regime of hydrodynamic lubrication, which allows for lateral movement of the piston (known as piston slap) during the combustion cycle. This phenomenon leads to the generation of high-frequency vibrations, hastening wear on the cylinder liner’s secondary thrust side. It also increases oil consumption due to inadequate sealing. In severe instances, it may result in blow-by gases tainting the crankcase oil, thus diminishing engine performance [48]. Conversely, an insufficient clearance (less than 50 μm) presents a risk of thermal seizure in high-load scenarios. This is because the steel piston expands at a rate of 12–14 × 10^−6^/°C, which is more rapid than the expansion of the gray cast iron cylinder liner at 11 × 10^−6^/°C. The noted increase in clearance, attributed to accumulated wear and thermal cycling [49], emphasizes the need for adaptive design strategies. Such strategies include graded thermal expansion coatings or the use of composite pistons to sustain optimal tolerances throughout prolonged service intervals.

In the methanol fuel engine test, the material compatibility between the CRGCI cylinder liner and the F38MnVS steel piston was influenced by several factors, including differences in thermal expansion characteristics, the corrosive environment, and the compatibility of the lubricating oil. The disparity in expansion coefficients 11 × 10^−6^/°C for gray cast iron and 12–14 × 10^−6^/°C for the steel piston necessitated a larger compensation allowance in the design of room temperature clearance. However, test data indicated that, after the endurance test, the cylinder–piston clearance generally exceeded the required range of 50–95 μm. This clearance further increased under hot working conditions, exacerbating the lateral movement of the piston. The acidic products, such as methanoic acid, generated by the combustion of methanol, accelerated the corrosion and wear of the gray cast iron cylinder liner. The maximum wear in the localized area of cylinder liner #1 reached 0.026 mm (as shown in Table S4), exceeding the tolerance by 4%. In addition, the corrosion and roughness on the surface of the cylinder liner deteriorated the boundary lubrication conditions. Coupled with the insufficient ability of 10W30 lubricating oil to resist methanol dilution and the reduction in oil film strength, the wear between the piston and the cylinder liner was exacerbated. The shrinkage of the piston skirt diameter exceeded the maximum allowable value of 95 μm as specified in the technical requirements, further increasing the fitting clearance between the cylinder liner and the piston. Test data confirmed that the current material combination of the cylinder liner and piston did not fully meet the enterprise’s technical requirements, resulting in a fitting clearance of the cylinder exceeding the reasonable range of 50–95 μm, thus making it challenging to meet the durability requirements of 3500 h under high load.

#### 3.3.2. Piston Ring Inspection

Table 9 and Table 10 present the test data for the first and second piston rings, respectively, where the primary measured parameters included the ring height, radial thickness, and closed gap of the piston rings. According to Table 9, the ring height of the first piston ring was 2.816 mm, with a permissible range of −0.03 to −0.01 mm, translating to an allowable range of 2.786–2.806 mm. The measured values for the ring height of piston rings numbered 1–6 ranged from 2.791 to 2.794 mm. These data exhibited high consistency, with a variation of only 0.003 mm, indicating uniform wear. The radial thickness measured 4.7 ± 0.15 mm. All recorded values, ranging from 4.72 to 4.74 mm, fell within the specified tolerance and were in close proximity to the nominal value of 4.7 mm. The minimal range of only 0.02 mm indicated that wear was manageable and that the first ring did not experience abnormal deformation or material loss. The clearance was measured at 0.25–0.35 mm, with all recorded values ranging from 0.28 to 0.30 mm, which also met the tolerance and were concentrated around the lower and middle limits. Furthermore, there was a margin of 5–14% from the upper limit of 0.35 mm.

Table 10 presents the detection data for the second piston ring, where the allowable range for ring height was between 2.47 and 2.49 mm. All measured values, ranging from 2.483 to 2.488 mm, fell within this tolerance and were close to the upper limit of 2.49 mm, exhibiting a range difference of 0.005 mm. The permissible range for radial thickness was 4.55–4.85 mm, and the measured values, which ranged from 4.70 to 4.73 mm, also complied with the tolerance requirements. Furthermore, the technical specifications for the closed gap were set between 0.50 and 0.70 mm, with the measured values ranging from 0.66 to 0.69 mm, thus satisfying the technical requirement tolerance. In accordance with the enterprise’s requirements for the design of piston ring structures, the dimensions of the three detection items for test piston rings 1–6 all fell within the specified design parameters. The data results indicated that during the 3500 h durability test, the DLC coating and PVD chrome plating applied to the piston rings met the operational requirements of the methanol engine.

## 4. Conclusions

Through a systematic investigation of the corrosion–wear coupling mechanisms in methanol-fueled engines, critical insights were gleaned regarding material selection and surface engineering strategies, with the aim of enhancing component durability. The following main conclusions were obtained.

CRGCI demonstrated optimal corrosion resistance in environments with low concentrations of formaldehyde and formic acid (formaldehyde ≤ 1.5%, methanoic acid ≤ 0.02%), with a corrosion depth of ≤0.055 μm. This was attributed to the synergistic effects of Cr/Sn/Cu microalloying in the pearlite matrix, which effectively suppressed micro-cell formation through multi-element passivation. The directional distribution of flake graphite additionally reduced continuous matrix damage by limiting the spread of localized corrosion. Moreover, the dynamic reconstruction of the Cr_2_O_3_-Fe_2_O_3_ composite passive film in HCCI identified a critical mechanism for enhancing corrosion resistance in high-concentration environments.

The DLC coating displayed remarkable corrosion resistance, demonstrating only 11.7% of the weight loss observed in CKS coatings under high-concentration conditions. Its amorphous structure effectively obstructed the permeation pathways of formate ions while simultaneously reducing abrasive wear due to its inherent lubricity. Comparative analyses showed that the compatibility of materials between components significantly influenced long-term performance. The mismatch in thermal expansion coefficients between F38MnVS steel pistons (12–14 × 10^−6^/°C) and gray cast iron cylinder liners (11 × 10^−6^/°C) resulted in an excessive clearance of 105 μm after 3500 h of operation, surpassing the permissible limit of 95 μm. This result emphasizes the need for coordinated thermal design in methanol engine components.

Bench testing validated the synergistic advantages of the HCCI-DLC combination, which reduced TDC wear by 67.7% (from 17.152 μm to 5.537 μm) and BDC wear by 68.5% (from 4.244 μm to 1.337 μm) compared with CRGCI. At the push side, the TDC wear reduction reached 80.1%, demonstrating HCCI-DLC as the optimal material-coating combination for methanol engines under high-load conditions. This configuration maintained cylinder/piston clearance within 50–95 μm (vs. CRGCI’s 84–105 μm), ensuring compliance with technical specifications. The results indicate a collaborative paradigm between materials and coatings that effectively addresses both corrosive degradation and mechanical wear in methanol combustion environments. For commercial engines operating under full-load conditions, such a reduction could translate to a service life increase from 8000 to over 40,000 h before overhaul, where wear depths exceeding 50 μm typically necessitate component replacement [50]. This outcome aligns with the durability targets set by marine and heavy transport sectors adopting methanol fuel [51].

For engine designers, these results emphasize the critical role of material-coating synergies in methanol-fueled systems. The HCCI-DLC configuration, validated under full-load bench conditions, is directly applicable to marine and heavy-duty engines where the corrosive byproducts of methanol and high thermal stresses are common. Scaling this solution for industrial production necessitates optimizing chromium content in HCCI (25–30% Cr) to balance cost and performance. High-throughput PVD processes should be established for DLC deposition to ensure economic viability. Future research should prioritize evaluating long-term stability under variable load cycles and developing adaptive coatings to optimize the balance between corrosion and wear resistance across operational temperature ranges.

## Figures and Tables

**Table 1:** Chemical composition of the cylinder liners (wt%).

Element	C	Si	Mn	Cr	P	S	Cu	Sn	Nb	Mg	Ti	B
CRGCI	1.7–2.8	2.4–4.0	≤0.7	0.2–0.8	≤0.1	≤0.08	0.1–0.5	0.05–0.2	0.1–0.7	—	—	0.02–0.08
HCCI	0.5–3.5	2.5–4.0	≤0.5	25–30	≤0.1	≤0.05	≤0.1	—	—	—	≤1.0	—
NCI	3.0–4.0	2.0–3.0	0–0.35	0–0.4	0–0.08	0–0.03	—	—	—	0.03–0.08	—	—

**Table 2:** Chemical composition of the pistons (wt%).

Element	C	Si	Cu	Mg	Ni	Mn	S	P	V
ZL109	—	11.0–13.0	0.5–1.5	0.8–1.3	0.8–1.5	—	—	—	—
F38MnVS	0.35–0.42	0.3–0.8	≤0.3	—	≤0.3	1.2–1.6	0.035–0.075	≤0.035	0.08–0.15

**Table 3:** Ratio of corrosive solution (wt%).

No.	Formaldehyde	Methanoic Acid	Lubricant
#1	0.5	0.01	99.49
#2	1.5	0.02	98.48
#3	2.5	0.10	97.40

**Table 4:** Basic parameters of the methanol engine test.

Parameters	11 L Engine	13 L Engine
Compression ratio	12.5:1	12.5:1
Power rating (kW)	260 (2300 r/min)	301 (1900 r/min)
Maximum torque (N·m)	710 (1300–1500 r/min)	1800 (1100–1400 r/min)
Minimum fuel consumption rate (g·(kW·h)^−1^)	≤470	≤450

**Table 5:** Average corrosion depths with standard deviation of the cylinder liner specimens in the three corrosive solutions.

No.	Formaldehyde/Methanoic Acid (%)	CRGCI (μm)	NCI (μm)	HCCI (μm)
#1	0.5/0.01	0.042 ± 0.005	0.128 ± 0.012	0.105 ± 0.008
#2	1.5/0.02	0.055 ± 0.006	0.242 ± 0.018	0.352 ± 0.022
#3	2.5/0.10	0.591 ± 0.035	1.241 ± 0.045	0.232 ± 0.015

**Table 6:** Average corrosion depths for both piston materials (μm).

Materials	Density (mg/mm^3^)	Volume (mm^3^)	Pre-Test Weight (mg)	#1	#2	#3
F38MnVS	7.85	1875	14,718.75	−0.155 ± 0.02	−0.166 ± 0.03	−0.676 ± 0.04
ZL109	2.68	1875	5025	+0.746 ± 0.05	+1.622 ± 0.05	+1.201 ± 0.04

Note: + represents an increase in depths, and − represents a reduction in depths.

**Table 7:** The average wear depths with standard deviation of CRGCI and HCCI cylinder liners after 1500 h (μm).

Material	Average Wear Position	Front Side	Secondary Push Side	Rear Side	Primary Push Side	Average Wear	Reduction vs. CRGCI
CRGCI	TDC	13.077 ± 1.2	15.087 ± 1.8	17.114 ± 2.1	23.330 ± 3.4	17.152 ± 2.1	
	BDC	2.532 ± 0.3	6.133 ± 0.9	6.898 ± 1.1	1.414 ± 0.2	4.244 ± 0.6	
HCCI	TDC	1.227 ± 0.1	10.678 ± 1.2	0.765 ± 0.1	9.480 ± 1.0	5.537 ± 0.6	67.7%
	BDC	1.378 ± 0.2	1.068 ± 0.1	1.443 ± 0.2	1.458 ± 0.2	1.337 ± 0.2	68.5%

**Table 8:** Clearance between the piston and cylinder liner after 3500 h of testing (μm).

Parameter	Cylinder Liner	Piston Skirt	Cylinder Liner and Piston Clearance
	Y-Y	Y-Y	Y-Y
Maximum dimensional variation	+26	−75	105
Minimum dimensional variation	+9	−80	84
Range of technical requirements	0–25	−70 to –50	50–95

**Table 9:** First ring 3500 h test data (mm).

No.	Test Items	#1	#2	#3	#4	#5	#6
1	Ring height 2.816 (−0.03 to −0.01)	2.791	2.792	2.792	2.791	2.794	2.794
2	Radial thickness 4.7 ± 0.15	4.72	4.73	4.72	4.73	4.74	4.73
3	Closed gap 0.25–0.35	0.29	0.29	0.28	0.28	0.30	0.30

**Table 10:** Second ring 3500 h test data (mm).

No.	Test Items	#1	#2	#3	#4	#5	#6
1	Ring height 2.5 (−0.03 to −0.01)	2.485	2.488	2.487	2.483	2.488	2.488
2	Radial thickness 4.7 ± 0.15	4.72	4.73	4.71	4.71	4.70	4.70
3	Closed gap 0.50–0.70	0.67	0.68	0.67	0.66	0.66	0.69

## Data Availability

The original contributions presented in this study are included in the article. Further inquiries can be directed to the corresponding authors.

## Supplementary Materials

The following supporting information can be downloaded at: https://www.mdpi.com/article/10.3390/ma18091966/s1, Table S1: CRGCI cylinder liner bore wear after operating for 1000 h (μm); Table S2: CRGCI cylinder liner bore wear after operating for 1500 h (μm); Table S3: HCCI cylinder liner bore wear after operating for 1500 h (μm); Table S4: Clearance between the piston and cylinder liner after 3500 h of testing (μm).

## Nomenclature

CRGCI	Corrosion-resistant gray cast iron	TDC	Top dead center
HCCI	High chromium cast iron	BDC	Bottom dead center
NCI	Nodular cast iron	DLC	Diamond-like carbon
PVD	Physical vapor deposition	CKS	Chrom–Keramik–Schicht
