# Supplementary material for: Study on the Corrosion and Wear Mechanism of a Core Friction Pair in Methanol-Fueled Internal Combustion Engines
Source: Materials (Basel). 2025 Apr 25;18(9):1966. doi: 10.3390/ma18091966 (PMC12072919; doi:10.3390/ma18091966)
Supplement: Supplementary file 1 [file materials-18-01966-s001.zip › materials-3577870-supplementary.pdf]

Supplementary Tables

Table S1. CRGCI cylinder liner bore wear after operating for 1000 h (μm).

No.	Front Side	Secondary Push Side	Rear Side	Primary Push Side
#1	2.424	32.684	4.416	10.043
#2	1.299	30.056	5.022	8.398
#3	3.636	25.130	6.234	23.182
#4	3.290	17.403	7.273	8.485
#5	1.385	26.320	3.290	8.485
#6	2.944	31.558	9.610	7.100

Table S2. CRGCI cylinder liner bore wear after operating for 1500 h (μm).

No.	Position	Front Side	Secondary Push Side	Rear Side	Primary Push Side	Average Wear
#1	TDC	11.688	1.299	15.152	1.407	7.387
	BDC	1.19	1.407	0.866	1.299	1.191
#2	TDC	0.866	12.771	4.545	33.896	13.020
	BDC	2.922	1.364	8.571	1.169	3.507
#3	TDC	6.429	47.619	1.948	40.26	24.064
	BDC	5.195	16.883	2.078	1.299	6.364
#4	TDC	13.506	13.766	44.935	2.338	18.636
	BDC	1.039	5.195	11.948	2.338	5.130
#5	TDC	43.377	2.338	34.805	2.078	20.650
	BDC	1.818	2.857	16.104	1.558	5.584
#6	TDC	2.597	12.727	1.299	60.000	19.156
	BDC	3.03	9.091	1.818	0.823	3.691
Average wear at TDC		13.077	15.087	17.114	23.330	17.152
Average wear at BDC		2.532	6.133	6.898	1.414	4.244

Table S3. HCCI cylinder liner bore wear after operating for 1500 h (μm).

No.	Position	Front Side	Secondary Push Side	Rear Side	Primary Push Side	Average Wear
#1	TDC	1.385	12.727	0.519	10.649	6.320
	BDC	1.385	1.126	1.558	0.866	1.234
#2	TDC	1.126	11.342	0.952	11.082	6.126
	BDC	1.558	0.779	1.645	1.385	1.342
#3	TDC	0.606	8.398	0.606	10.043	4.913
	BDC	1.558	1.126	1.039	2.771	1.624
#4	TDC	1.645	10.909	0.606	10.216	5.844
	BDC	0.909	1.299	1.472	1.126	1.202
#5	TDC	1.039	12.468	1.039	9.351	5.974
	BDC	1.385	0.952	1.385	1.385	1.277
#6	TDC	1.558	8.225	0.866	5.541	4.048
	BDC	1.472	1.126	1.558	1.212	1.342
Average wear at TDC		1.227	10.678	0.765	9.480	5.537
Average wear at BDC		1.378	1.068	1.443	1.458	1.337

Table S4. Clearance between the piston and cylinder liner after 3500 h of testing (μm).

No.	Measurement Cross-Section	Cylinder Liner	Piston Skirt	Cylinder Liner and Piston Clearance
		Y-Y	Y-Y	Y-Y
#1	1B	+25	−75	100
	1C	+26	−75	101
#2	2B	+10	−76	86
	2C	+21	−76	97
#3	3B	+17	−75	92
	3C	+25	−75	100
#4	4B	+15	−79	94
	4C	+20	−79	99
#5	5B	+20	−80	100
	5C	+25	−80	105
#6	6B	+9	−75	84
	6C	+15	−75	90
Maximum dimensional variation		+26	−75	105
Minimum dimensional variation		+9	−80	84
Range of technical requirements		0–25	−70 to −50	50–95
